# Modeling the Dynamic of Multiwave Diseases: The Model of Hand, Foot and Mouth Disease

**DOI:** 10.3390/v16081217

**Published:** 2024-07-29

**Authors:** Patrice Ravel, Nghia Ngu Duy, Guilhem Kister, Le Thi Song Huong, Ankit Dwivedi, Christian A. Devaux, Tran Nhu Duong, Nguyen Tran Hien, Laurent Gavotte, Emmanuel Cornillot, Roger Frutos

**Affiliations:** 1 Institut de Recherche en Cancérologie de Montpellier (INSERM U1194), Université de Montpellier, CEDEX 5, 34298 Montpellier, France; patrice.ravel@umontpellier.fr (P.R.); adwivedi@som.umaryland.edu (A.D.); emmanuel.cornillot@umontpellier.fr (E.C.); 2 National Institute of Hygiene and Epidemiology, Hanoi 11611, Vietnam; ndn@nihe.org.vn (N.N.D.); trannhuduong@gmail.com (T.N.D.); ngtrhien@yahoo.com (N.T.H.); 3 Faculty of Pharmacy, University of Montpellier, 15 Av Charles Flahault, CEDEX 5, 34093 Montpellier, France; guilhem.kister@umontpellier.fr; 4 Hai Phong Preventive Medicine Center, Hai Phong City 180000, Vietnam; nguduynghia@gmail.com; 5 IHU Méditerranée Infections, CEDEX 5, 13385 Marseille, France; christian.devaux@mediterranee-infection.com; 6 Espace-Dev, University of Montpellier, CEDEX 5, 34093 Montpellier, France; laurent.gavotte@umontpellier.fr; 7 Cirad, UMR 17, Intertryp, TA-A17/G, Campus International de Baillarguet, CEDEX 5, 34398 Montpellier, France

**Keywords:** hand foot mouse disease, Hải Phòng, epidemiology, multi-scale analysis

## Abstract

An HFMD outbreak spread over the city of Hải Phòng from summer 2011 to autumn 2012. This epidemic was chosen because it was the very first HFMD epidemic in North Vietnam, eliminating thus interferences with previous outbreaks. This epidemic displayed three separate waves. A complete dataset was collected for more than 9500 patients during this period, which enabled us to analyze this epidemic at different scales. Access to the healthcare system was crucial during this period, which was possible due to a reorganization of the system in February–March 2012. An analysis at the commune level enabled us to track the epidemic along certain communication routes. The three-waves structure reveals a wide disparity at the district level. We developed a mathematical model showing high accuracy at the adjustment of data for both the total number of cases and for the number of cases per week. As a consequence, the model was able to accurately determine the dates of the beginning and end of each wave and to show that they overlapped. Using mathematical functions associated with this model, it was possible to calculate the probability for a patient to belong to a specific wave.

## 1. Introduction

Hand, foot and mouth disease (HFMD) is often a multiphase disease involving a succession of different viruses and waves. The co-circulation of different serotypes and alternation between enterovirus A71 (EV-A71) and coxsackievirus A (CV-A) was commonly observed during HFMD epidemics [1,2,3,4]. However, co-infection is not a primary cause of severe forms [5,6]. Epidemics could be due to both the accumulation of susceptible individuals in the community, since patients are mostly young children under five, and the introduction of new genotypes or strains [7]. Within the West Pacific–South East Asia region, outbreaks in Taiwan in 1998 and 2000 were caused by EV-71 C2 and B4 strains, respectively [8]. Sentinel surveillance in Sarawak, Malaysia, demonstrated that the emergence of the novel subgenotype C1 of EV-71 was the cause of the 2003 outbreak [9]. Although very often mild, HFMD can result in severe complications such as encephalitis, aseptic meningitis, pulmonary edema, myocarditis, and death [10], and it can be devastating like in Cambodia in 2012, where there was a death rate of 88% [11]. This large panel of symptoms and variation over time depend for a good part on the succession of viruses driving the epidemics. Since this early period, the incidence rate of HFMD in the Asia–Pacific region has been increasing, and China is the main endemic area [12].

A relevant issue in HFMD epidemiology could be to understand and distinguish the different waves during a multipeak epidemic. Waves may be associated with different viruses with different transmissibility and virulence characteristics. Understanding the process of emergence of a disease can be related to the ability to identify type samples from outliers. Dynamic models are therefore required for analyzing epidemics. However, if current models can analyze individual waves, there is still a need to model and compute a multiwave epidemic. The 2011–2012 HFMD epidemic was the largest experienced in Vietnam and the very first one to occur in North Vietnam. Hải Phòng displayed the highest prevalence in North Vietnam, providing thus a large cohort of more than 9000 patients to analyze (Supplementary Table S1). Being the first HFMD epidemic in North Vietnam, this outbreak provided an opportunity to study the dynamic without interference from previous outbreaks. We thus report here a mathematical approach for the discrimination of individuals within an outbreak time frame and a mathematical model for characterizing multiwave outbreaks displaying differing waves associated with virulence or transmissibility [13].

## 2. Material and Methods

### 2.1. Epidemiological Information and Specimen Collection

All HFMD cases in Hải Phòng province were reported to the National Institute of Hygiene and Epidemiology (NIHE) through the national communicable disease surveillance system since 2011.

### 2.2. Statistical and Mathematical Analysis

Mean comparison was implemented by Student’s *t*-test. A Chi-square test was used for proportion comparison of the Hải Phòng city population, and a one-way ANOVA test was used for the variance analysis. The Earth Mover’s Distance (EMD) distance was calculated using the emd (F1, F2, W1, W2, Func) function in Matlab v8. GIS analysis was performed using QGIS v2.

### 2.3. Bias and Ethics

Training session HFMD cases definition and reporting were organized for the staff of the routine surveillance system to enhance the quality and consistency of case reports. This work was conducted following the requirements of the Vietnamese Ministry of Health and under the Law of Communicable Diseases Prevention and Control passed in 2007.

### 2.4. Spatial Heterogeneity of Hải Phòng District

**Figure 1:**
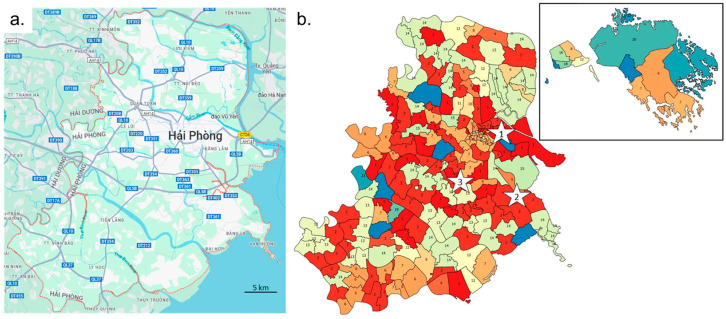
Spread of HFMD disease along communication routes. (**a**) Main roads and waterways in the Hải Phòng region. Source: Google Maps. (**b**) Classification of communes according to the temporal distribution of HFMD cases over the 2011–2012 period. Clusters with the same or close colors have a similar distribution of cases over the period of the three epidemic waves (cluster ID is given in each commune). Stars indicate the estimation site of emergence for each wave. Six communes were rejected for having an insufficient number of cases.

The spatial organization of the Hải Phòng province could have played a major role in the dynamic of expansion and evolution of the disease. HFMD transfer depends both on natural and anthropogenic environment parameters. The main factors are rivers, roads and settlements. The main west–east orientation of the rivers limits the north–south circulation, whereas west–east transit between the coast and mainland is important (Figure 1a). Main road networks are located a distance from the main rivers but follow the same main west–east orientation. North–south connections are less common and correspond to few secondary roads and rivers. The density of secondary roads increases strongly in the Hải Phòng city suburb area. Settlements are generally isolated from the main rivers, mostly because of flood risk. Rural areas are connected to settlements by province roads yielding a good locally connected settlement network, but they remain fragmented as pockets isolated by the main rivers from neighboring territories. Hải Phòng city and its suburb area sprawl west–east without crossing the river to the north. Settlement clusters south of Hải Phòng are regularly spread, whereas on the northern part of the province, high concentrations are found along the main country road, while villages are scattered over the northern territory (Supplementary Figure S1). Correspondence of communes, ID numbers, district names and commune names are given in Supplementary Table S2.

## 3. Results

### 3.1. HFMD Burden during the 2011–2012 Epidemic

**Figure 2:**
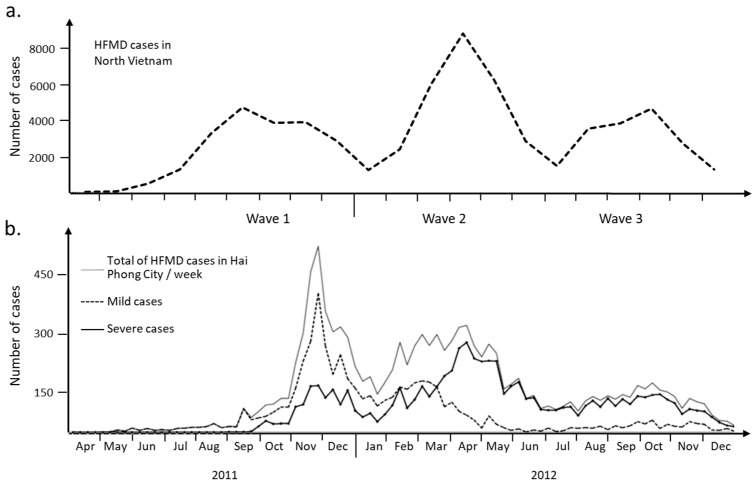
Evolution of HFMD cases over the epidemic period (2011–2012). (**a**) Monthly report of HFMD cases in North Vietnam. Number of HFMD cases reported to Vietnam Ministry of Health as routine surveillance in 2011 and 2012. (**b**) Weekly HFMD cases and severity distribution in Hải Phòng province. Each epidemiologic week begins on Monday. Mandatory reporting of the disease began in 2011. Mild cases are cases free of complication. Severe cases are characterized by febrile exanthemata’s symptoms affecting the central nervous system, frequently myoclonus and more severe neurological complications.

A large HFMD epidemic hit North Vietnam in 2011–2012 (65,949 cases, source: General Department of Preventive Medicine, Ministry of Health). It was the first outbreak recorded in this part of the country. The region of Hải Phòng was the hardest hit among the 28 North Vietnam provinces (Figure 2a). The epidemic in Hải Phòng province was slightly delayed in 2011 when compared to the rest of North Vietnam. The index case was a 6-year-old girl admitted in an urban district hospital on 19 April 19 2011 (delay of 2 days between the onset of symptoms on 17 April and admission at the district hospital, Supplementary Table S1). In total, 9621 cases were collected that could be of three waves during the time of the epidemics (Figure 2b). Several changes happen during the period of study that must be clarified. A first guideline concerning the surveillance, prevention and control of HFMD was published on the 24 February 2012. The second guideline about diagnosis and treatment was issued on 30 March 2012. The publication of these two specific guidelines by the Ministry of Health was necessary to improve the management of patients with severe signs of the disease. The influx of patients of all types to hospital led to overcrowding of the healthcare system during the first phase. The health system was reorganized during the second phase of the epidemic to deal only with the most severe cases. The result was an inversion of the curves between severe cases and those of lesser severity (Figure 2b). The etiological agent is a second element that needs to be taken into account in the overall picture of this epidemic in the Hải Phòng region. The first two phases of the epidemic were mostly associated with enteroviruses—mainly EV-71 according to molecular analysis performed on 257 throat swabs collected at the main pediatric hospital in Hải Phòng city. Wave 3 was associated with the co-circulation of CV-A6 and CV-A16.

The early cases appeared in the northern and central part of the region i.e., Hải Phòng city (Supplementary Figure S2). The first cases emerged in the district of Ngô Quyền before spreading south, west and later northwest. The southern part of the province occurrence of subsequent cases was strongly delayed. Only one early location was found in the southern part, corresponding to the largest urban area, while two locations are identified in the southeastern coastal region. Early cases were also recorded on some commune of the main island. The main axis of virus expansion in the north is primarily west–east, spreading over the northern part of the province along rivers and the main country roads. The second wave started in the more southern district of Dương Kinh and then spread south and west before going back to the east. Finally, the third wave started in the central district of Kiến An and followed a similar route as wave 2.

### 3.2. Local Heterogeneity of the Epidemic

A film summarizes the number of cases over time in each commune (Supplementary Video S1). Foci of infection emerged sporadically. The frequency of variations in each commune increases during the epidemic waves. Some nearby communes appear to behave in a coordinated fashion or just spread out over time, suggesting possible transmission routes for the virus in the region. The EMD (Earth Mover’s Distance) method was used to compare the case distribution between communes. The number of cases was reported by month for each commune to obtain enough data for a low-density commune. A distance matrix of all communes was used to build a tree and a classification based on 21 clusters that were then mapped on the communes (Figure 2b). Virus circulation followed some communication routes. The red communes (cluster 1 and 2) in the center of the region are along the west part of the QLB5 main road and a secondary route from the main crossroad in the Hồ Nam commune of Lê Chân district. They correspond to the east-to-west transmission of the virus from the sites of emergence of waves 2 and 3. Cluster 2 was also found in the southwest part of the region for communes located along QL10 road. District level confirmed the heterogeneity of the epidemic in the region (Supplementary Figure S3). Most districts show three epidemic waves. The intensity of each wave varies from district to district. The most populous and popular urban districts show a distribution similar to that observed across the region as a whole. There is also a difference in patient management and adherence to guidelines published in February and March 2012 in one district in the south of the region and one urban district in the east. Local heterogeneity and changes of guidelines question the possibility of modeling the epidemic in three waves with a unique model.

### 3.3. Model Development

The in-depth characterization of the epidemic showed that within the one-year period of the study, three different waves occurred, each separated by a minimum, i.e., a drop in the number of cases followed by an increase. This in-depth analysis also showed that the diversity of symptoms is not a criterion for characterizing a wave, as it is dependent on parents and physician behavior applying a precautionary attitude or possibly exaggerating the symptoms to ensure an immediate care. Preliminary analyses suggest that the evolution of the epidemics could be described with a Bernoulli differential equation for each wave whose solution is a logistic function. Each wave was analyzed separately to find objective parameters of the model. In a first step, we assumed that each wave is separated from the other. Then, in a second step, the model is generalized for the three waves, and the parameters are optimized for taking into account the potential overlap of the waves. This means that a wave is closing when the next wave is starting in a sense to be specified later. The logistic function was therefore aiming at describing HFMD cases over time.

The total number of HFMD cases for one wave is N. Over a defined period called h, the number of new cases depends of the number of susceptible patients in the population *X_s_*(*t*) and two parameters related to the epidemic: the probability K(t) to be exposed to the virus and the K_0_ interaction factor, which is the probability of presenting clinical symptoms. These two probabilities reflect the virus-associated parameters, which are virulence and pathogenicity, respectively.

The cumulative number *X_m_*(*t* + *h*) of patients is governed by the equation for one wave:

(1) $$X_{m}\left(t+h\right)=X_{m}\left(t\right)+K_{0}K\left(t\right)X_{s}\left(t\right)h,\;t\geq t_{0}$$

with

$$X_{s}\left(t\right)=N-X_{m}\left(t\right)\;\mathrm{and}\;K\left(t\right)=\frac{X_{m}\left(t\right)}{N},$$

The dot K(t) X_s_(t) is the expected number of exposed patients. The constant K_0_ is called the interaction factor and is the probability of an exposed patient becoming sick.

By passing to the limit as h tends to 0, we obtain a differential equation of Bernoulli (2)

(2) $$\frac{X_{m}^{\prime }\left(t\right)}{X_{m}^{2}\left(t\right)}-K_{0}\frac{1}{X_{m}\left(t\right)}+\frac{K_{0}}{N}=0,\forall t\geq t_{0}\;\mathrm{and}\;X_{m}\left(t\right)\neq 0$$

with the two limits conditions:

The general solution of Equation (2) is the well-known logistic function defined by three parameters (K_0_, K_1_, K_2_), K_1_ and K_2_ being defined by the exact integration of Equation (2):

(3) $$X_{m}\left(t\right)=\frac{K_{2}}{\left(1+\exp\;(-K_{1}-K_{0}(t-t_{0}))\right)},\forall t\geq t_{0}$$

With the help of the vector ***Y*** of observed values ${Y\left(t_{i}\right)}_{0\leq i\leq k}$, the constants K_0_, K_1_, and K_2_ are computed with a Jacquard algorithm by minimizing

(4) $$Ec\left(K_{0},K_{1},K_{2}\right)=\sum_{i=0}^{\tau }{\left(y\left(t_{i}\right)-X_{m}\left(t_{i}\right)\right)}^{2}$$

Note that τ is the experimental end of the wave. This corresponds to the time of the last observed case of a given wave. The time t_0_ is the date of the first case observed for a given wave. Nevertheless, Equation (3) can be interpreted with two limits conditions:

$$\underset{t\to +\infty }{\mathrm{Lim}}\;X_{m}\left(t\right)=N\;and\;X_{m}\left(t_{0}\right)=N_{0}$$

N and N_0_ are theoretical values. They are, respectively, the total number of cases at the theoretical end of the waves t=+∞ and the number of cases at the beginning of the wave. With Equation (3), we have N = K_2_ and $N_{0}=\frac{K_{2}}{\left(1+\exp(-K_{1})\right)}$.

Owing to the limit conditions and N > N_0_, it is possible to demonstrate that the interaction factor K_0_ is positive and less than 1.

### 3.4. General Model and Overlapping Waves

**Figure 3:**
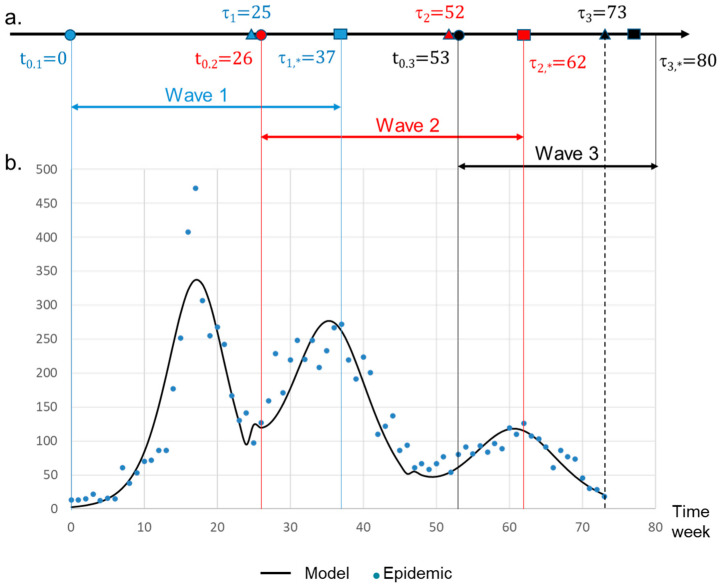
Derivative mathematical model of the 2011–2012 HFMD epidemic dynamic. (**a**) Timeline parameters. The theoretical curve is obtained by the derivative of the function *X_G_*(*t*). Beginning of a wave is labeled t_0_. The time τ corresponds to the last known case for a given wave. The time τ_*_ is an estimation of τ obtained by the model (Equation (3)). Beginning (t = 0 in the model) of wave 1 was set at week 16 (Supplemental Table S1). (**b**) Evolution of the number of cases per week. Dots are associated with the observed values. The solid curve is the theoretical number of cases.

We assumed that the time of the beginning of each wave (t_0_) is known. Therefore, the beginning of the wave 2, $t_{0.2}$, occurred after the date of the last observed case of wave 1 $\tau_{1}$. The same condition is assumed for wave 2 and wave 3. This means that $t_{0.2}>\tau_{1}\;\mathrm{and}\;t_{0.3}>\tau_{2}$ (Figure 3a). These dates are provided by physicians and clinical files. The theoretical time of the end of a wave is therefore t = +∞. The parameter τ_*_ is an underestimation of this date. It is defined as $\tau_{*}=inf\left\{t/\left|Xm(t)-N\right|<\epsilon \right\}$. To compute values (K_0_, K_1_, K_2_) of Equation (3), we first began with wave 1 using the Jacquard algorithm (Table 1). The end of wave $\tau_{1*}$ was estimated to occur at week 37. For wave 2, the observed frequencies were therefore corrected between the following dates: the beginning of the wave 2: $t_{0.2}=26$ and $\tau_{2}=52$ (numbers express weeks). The correction consisted in subtracting wave 1 frequencies from those observed for wave 2 over the overlap period. The corrected frequencies allowed thus to take into account the overlapping of the two subsequent waves before applying the Jacquard algorithm to wave 2 (see Table 1). The end of wave 2 (τ_2,*_) was estimated at week 62. As the observed frequencies were underestimated compared with the mathematical model (Figure 3b), no correction was necessary for wave 3. The computed parameters for wave 3 are given in Table 1. For all the waves, the quality of fit was high with a coefficient of determination R^2^ higher than 0.997. The computation of the general model $X_{G}\left(t\right)$ is obtained by the summation of the three logistic function (3), and the theoretical total number of cases at time *t* is given by

(5) $$X_{G}\left(t\right)=X_{m,1}\left(t\right)I_{[0,+\infty [}+X_{m,2}\left(t\right)I_{[26,+\infty [}+X_{m,3}\left(t\right)I_{[53,+\infty [},\forall t\geq t_{0}$$

$I_{[a,+\infty [}=1\;if\;t\in [a,+\infty [,\;o\;if\;t\notin [a,+\infty [$ during the three waves is shown in Supplementary Figure S4.

### 3.5. Parameters of Interaction between Disease and Environment

The K_0_ parameter represents the interaction factor during each wave (Table 1). K_0_ can be compared with the R0 basic reproduction number, which estimates the speed at which a disease is capable of spreading. Wave 1 was significantly (*p* < 0.05) the most virulent, which was followed by wave 3 and wave 2 in decreasing order. These results are in agreement with the literature. EV-71 present a reproductive number (R0) close to five whether it was estimated to 2.5 for CV-A16 [13]. With respect to patient classification, the definition of X_m_(t) (Equation (3)) applied to a wave W makes it possible to build the probability for an individual to belong to a specific wave W. Indeed, with a specific constant A

(6) $$f_{w}\left(t\right)=\frac{1}{A}X_{m}\left(t-t_{0}\right),\forall t\geq t_{0}\;\mathrm{with}\;A=\int_{t_{0}}^{\tau }X_{m}\left(t-t_{0}\right)dt$$

f_w_(t) is a density probability. So

(7) Pt0≤u≤TW=∫t0Tfwt−t0dt,

is the probability to have an inclusion time between [t_0_, T] when a patient belongs to a given wave W. It is therefore possible to extract patients belonging to a specific wave with a certain probability and then to express the probability for an individual to belong a specific wave.

As a result, 90% of the patients were associated to a single wave. We could confirm that the severity factor and the wave factor are significantly correlated (*p* < 0.00001). The percentage of severe cases is less important in wave 1 compared to the others, which is the result of guidelines modification. The possibility of modelizing wave 2 similarly to wave 1 and wave 3 confirms the positive effect of guidelines modification, as more attention was paid to severe patients [13,14]. We notice that the age distribution shifted on younger patients in wave 3 compared to the others (*p* < 0.0001). This difference could be related to the etiological agent of the disease.

## 4. Discussion

We question the representation of the epidemic at individual, commune, district and region scales. Figure 2b shows that the three waves can be heterogenous in terms of cases severity. The guidelines modifications published in 2012 emphasize an important point in epidemiology: an individual is taken into account only when it is recorded by the health system. We did observe some local variation, but the guidelines were very well observed, and the health system’s response was rapid, even though it took place in the middle of wave 2. The multiscale study enabled us to show how the epidemic unfolded over the 2011–2012 period. The first wave arrived from the north (Supplementary Figure S1). The main focus of emergence remains the city center of Hải Phòng. Waves 2 and 3 emerged in urban areas, but they were further south in the Lê Chân and Hai An districts. These two waves followed a similar diffusion route, which was marked by clusters 1 and 2 of communes obtained by comparing monthly distributions of the number of cases (Figure 2). The local heterogeneity motivated the search for a general method to model over time the multiwave epidemic observed at scale of the Hải Phòng region. The question was addressed specifically to wave 2. In addition to the guidelines modification, wave 2 was also characterized by a high level (close to 50%) of negative PCR, suggesting the presence of a specific etiological agent.

The mathematical model developed in this work is based on the virulence/transmissibility constant, in other words, on differing dynamics of the host–virus interaction. The model is based on the principle that the key factor is not the overall number of patients but indeed the differing dynamic of host–virus interaction. Compiling the number of cases has the consequence of smoothing all data and to show the epidemic as unimodal. Regardless of its true nature, the epidemic is considered to be associated with a single causative agent. The model developed in this work enables the break down of the epidemic into its various components when they exist and displaying the epidemic as a multimodal curve. In this case, each mode corresponds to a specific agent displaying a specific dynamic.

The model demonstrates that three distinct waves occurred during the 2011–2012 Hải Phòng HFMD epidemic, each one displaying a specific dynamic of expansion. According to the K0 parameter, the level of host–virus interaction was different for each wave. Transmissibility was therefore a discriminating factor, as each wave displayed a different speed, and the highest transmissibility was associated with wave 1. Wave 2 displayed an intermediate speed, whereas wave 3 was characterized by the slowest speed of expansion. This result is in agreement with R0 values, which are higher for EV-71 than for CV-A6 and CV-A16 [15]. A first estimate of R0 can be obtained from the model: R0 = K_0_/(1/T) = K_0_T, where T is the average transmissibility time of HFMD. The recommended isolation period is 7 days. This value of T gives for wave 1, the most virulent one, R_0|1_ = 0.36 × 7= 2.5. This result should be treated with caution, as it does not take into account asymptomatic cases which are not included in this study. The situation is even worse for waves 2 and 3, during which the healthcare system was focused on managing the most severe cases following the publication of specific guidelines between February and March. Unlike COVID-19, we have no large-scale sequencing data to associate the K_0_ value with a particular virus. Recent pandemics have demonstrated the value of these types of data.

The model developed in this work also allowed for assigning the beginning and end of each wave with a highly significant fit with observed data. It was thus possible to show that the three waves partly overlapped, each wave starting before the beginning of the previous one. This indicates that the replacement of the causative agent occurs during the span of a given wave and not after this wave. It is therefore difficult to only explain this phenomenon by an adaptation of the human population through immune resistance. A hypothesis could be that each causative agent is capable of infecting only a specific, susceptible part of the human population and disappears when the available naive population falls below a given threshold. However, it is in this case difficult to explain why all viral strains do not expand at the same time, providing they do not target the same populations and why there is a pattern of successive waves. The relative virulence of each viral strain might therefore play a key role in particular in asymptomatic patients where competition between strains might occur. Nevertheless, this also suggests that all strains circulate at the same time and that a given strain will predominate and expand depending on the immune-resistance/transmissibility ratio. Although we cannot clearly explain the phenomenon, the model developed in this work can thus well describe the replacement of the virus as the epidemic progressed and accurately determine the start and end of each individual component of the epidemic.

Beyond that, the model is also capable of addressing the very important issue of calculating the probability of a patient to belong to a given wave and to classify the patients into specific clusters with a high level of confidence. These clusters can be associated with a specific typology of symptoms and dynamic traits. Doing so, the model allows for estimating during the span of the epidemic whether a new wave occurred before the peak of this new wave and to identify the patients affected by this new wave comparatively with patients affected by the previous wave. This in turn open ways for clinicians to determine if a new treatment strategy or a new crisis management is needed, and if so, it also provides the means of identifying the patients to be considered as a priority. The most important aspect is that this can be completed during the span of the epidemic. The model provides thus means to facilitate real-time actions.

Beyond the specific case of HFMD, the model described in this work can be applied to other diseases. If the disease considered is a single-phase, unimodal disease caused by a single agent, the model will bring nothing more than existing single-wave models. Similarly, if the disease is a multiphase, multimodal, disease caused by different agents but displaying the same virulence/pathogenicity traits, it can thus be considered similar to a single-wave disease, and here also, the model will bring nothing more than existing single-wave models. However, if the disease is a multiwave disease involving causative agents characterized by differing virulence/transmissibility traits, this model will bring very useful applications for managing this epidemic and for identifying an emerging wave associated with a potentially new strain. This work and the model presented can therefore bring very valuable support to public health in the management of multiwave infectious diseases. Indeed, it is to our knowledge the first time such a multiwave model with a very high fit with observed data capable of typing patients based on clinical description and determining the emergence of a new wave has been developed. Since it is not relying on molecular or serological tests but rather only on clinical parameters, the model is fast and easy to implement with no delay in response. The implementation of such a model on HFMD and other multiwave diseases will therefore bring valuable support in managing these important sanitary burdens.

## 5. Limitations

This work does not address the whole dynamic of HFMD in Vietnam but rather focuses only on one region, Hai Phong, during the 2011–2012 period. The rationale behind this study design was to develop a model describing the evolution of a multiwave viral disease. The 2011–2012 Hai Phong epidemic was the first one to have occurred in North Vietnam. Therefore, it was a perfect context to analyze the multiwave dynamic without interference from previous infection. HFMD occurred multiple times in South Vietnam and, therefore, this work cannot render the complexity of HFMD at the level of the whole country.

Another questionable aspect could be the lack of integration of asymptomatic patients. Such patients escape diagnosis and are not integrated in any clinical analysis. However, it is not a limitation, since the objective of the work was to design a model that could detect multiwave infections using clinical data only.

This article does not address very important potentially confounding factors such as changes in surveillance, reporting, and healthcare practices during the epidemic period, which could have impacted case numbers. These potentially confounding factors and the evolution of the epidemic are addressed in detail in a separate article [14].

COVID-19 has clearly shown the complex dynamic of viruses with the coexistence, selection and succession of multiple variants remaining often undetected at the clinical level. However, SARS-CoV-2 represents an exception. It is very closely monitored and systematically sequenced. The very high definition provided by sequence analyses allows for detecting minute differences and differentiating variants and lineages. However, these molecular differences do not always translate into a visible difference in epidemiology and clinic. This work cannot, and is not meant to, address this level of description of virus diversity. This work is aiming at discriminating variants and waves based on clinical differences, which is the level at which most viral epidemics are described and reported.

## Figures and Tables

**Table 1:** Characteristics of the three HFMD waves.

Wave	Parameter	Value	Standard Error
1	K_1_	−6.33	0.209
	K_0_	0.368	0.014
	K_2_	3673.91	67.64
2	K_1_	−2.541	0.075
	K_0_	0.259	0.009
	K_2_	4104.45	44.29
3	K_1_	−2.28	0.052
	K_0_	0.275	0.008
	K_2_	1746.58	21.29

K_0_ is the interaction factor. For wave 2, parameters displayed are the corrected parameters.

## Data Availability

All data have been made available in the manuscript and Supplementary Material.

## Supplementary Materials

The following supporting information can be downloaded at https://www.mdpi.com/article/10.3390/v16081217/s1. Figure S1: Land cover for Hai Phong District. Red. Urban areas. Artificial surfaces growth between 2000–2010 is in pink. Resolution 30 × 30 m. Figure 1 in grayscale has been superimposed on the map. Figure S2: Occurrence of HFMD index cases (t_0_) in each commune of the Hai Phong region. Colors represent interval of occurrence of the index case in each commune. Red is for the short time t_0_ and blue for a longer time. HFMD started in the center of Hai Phong City, then spread towards north west and after south west. Numbers represent Communes ID as given in supplemental Table S2. Figure S3: Monthly distribution of HFMD patients per district of the Hai Phong region. Level 1 correspond to non-severe cases according to guidelines from the Vietnamese Ministry of Health. Patients present only mouth ulcers and/or skin lesions. Higher level patients have additions symptoms. Patients at level 2b any higher are considered as severe cases in the present study (see legend of supplementary Table S1 for severity level definition). Figure S4: Temporal characteristics of the 2011–2012 HFMD epidemic. (**a**) Timeline parameters. t_0_ is the beginning of a wave. The time τ corresponds to the last known case for a given wave. The time τ_*_ is an estimation of τ obtained by the model (Equation (3)). Beginning (t = 0 in the model) of wave 1 was set at week 16 (supplemental Table S1). (**b**) Evolution of cumulative cases. Dots are associated to the observed values Y(t(i)). The solid curve is the theoretical number of cumulative cases *X_G_*(*t*). Table S1: Complete set of data for HFMD cases reported during the 2011–2012 period. Table S2: Correspondence of communes, ID numbers, district names and commune names. Video S1: Local heterogeneity of the epidemic. This film summarizes the number of cases over time in each commune. The EMD (Earth Mother Distance) method was used to compare case distribution between communes.
