# Supplementary material for: Modeling the Dynamic of Multiwave Diseases: The Model of Hand, Foot and Mouth Disease
Source: Viruses. 2024 Jul 29;16(8):1217. doi: 10.3390/v16081217 (PMC11359891; doi:10.3390/v16081217)
Supplement: Supplementary file 1 [file viruses-16-01217-s001.zip › viruses-3111778_suppl_proofed_final.pdf]

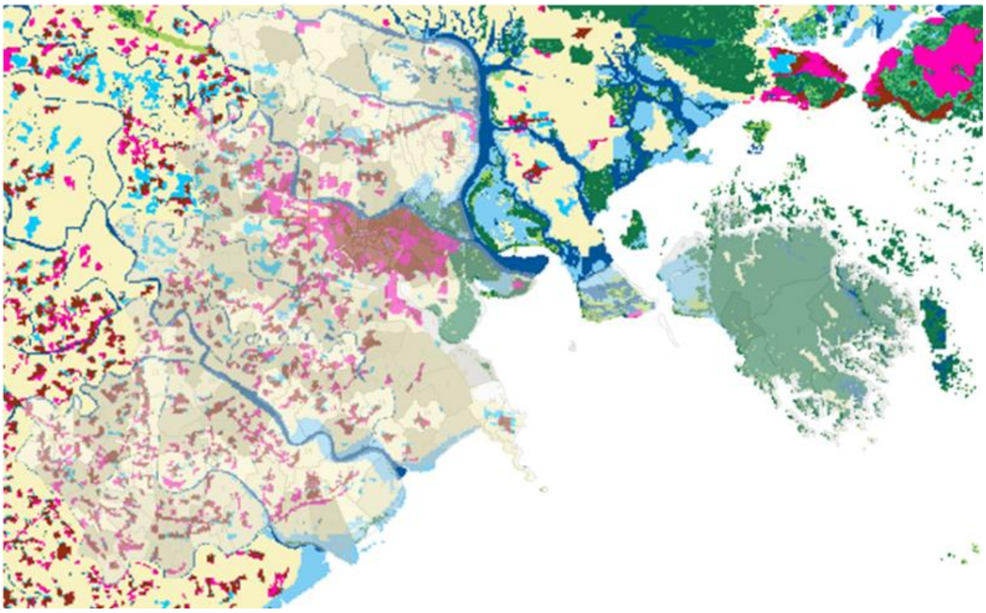

Figure S1. Land cover for Hai Phong District. Red. Urban areas. Artificial surfaces growth between 2000-2010 is in pink. Resolution 30 × 30m. Figure 1 in grayscale has been superimposed on the map.

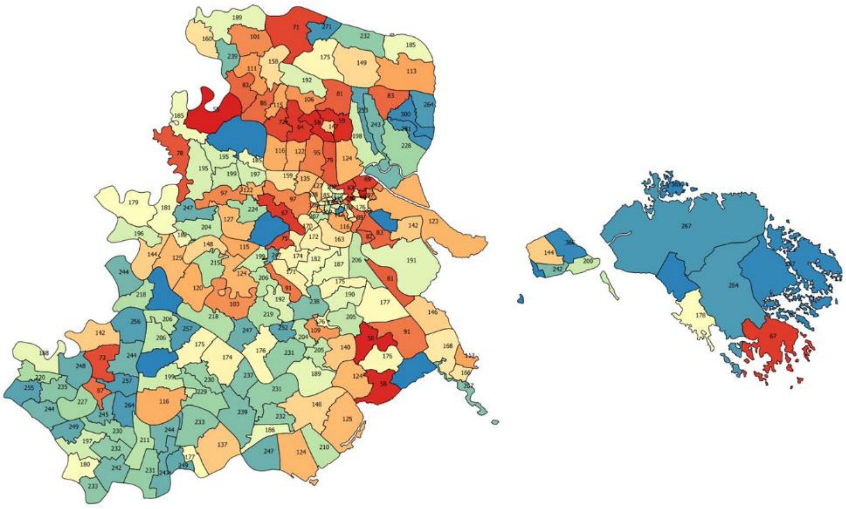

Figure S2. Occurrence of HFMD index cases (t0) in each commune of the Hai Phong region. Colors represent interval of occurrence of the index case in each commune. Red is for the short time t0 and blue for a longer time. HFMD started in the center of Hai Phong City, then spread towards north west and after south west. Numbers represent Communes ID as given in supplemental Table 2.

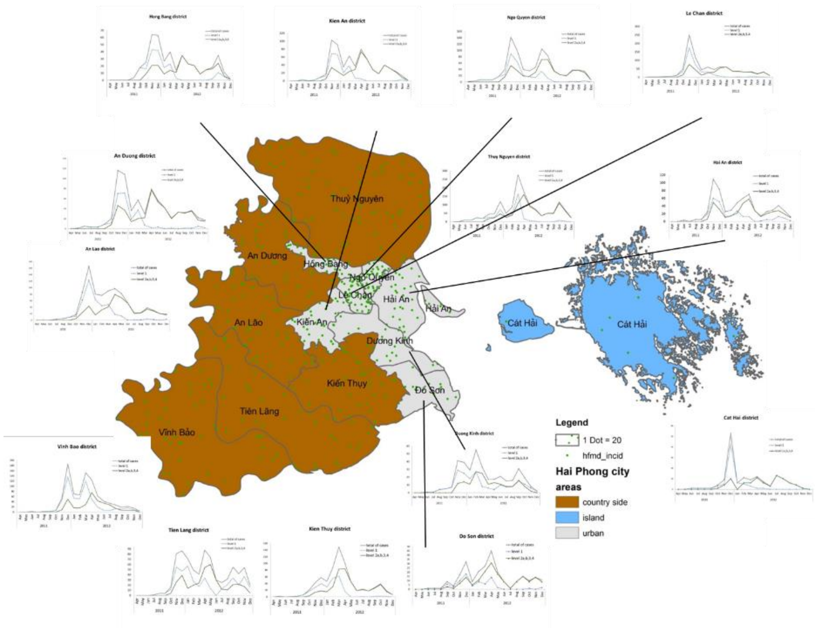

Figure S3. Monthly distribution of HFMD patients per district of the Hai Phong region. Level 1 correspond to non-severe cases according to guidelines from the Vietnamese Ministry of Health. Patients present only mouth ulcers and/or skin lesions. Higher level patients have additions symptoms. Patients at level 2b any higher are considered as severe cases in the present study (see legend of supplementary Table 1 for severity level definition).

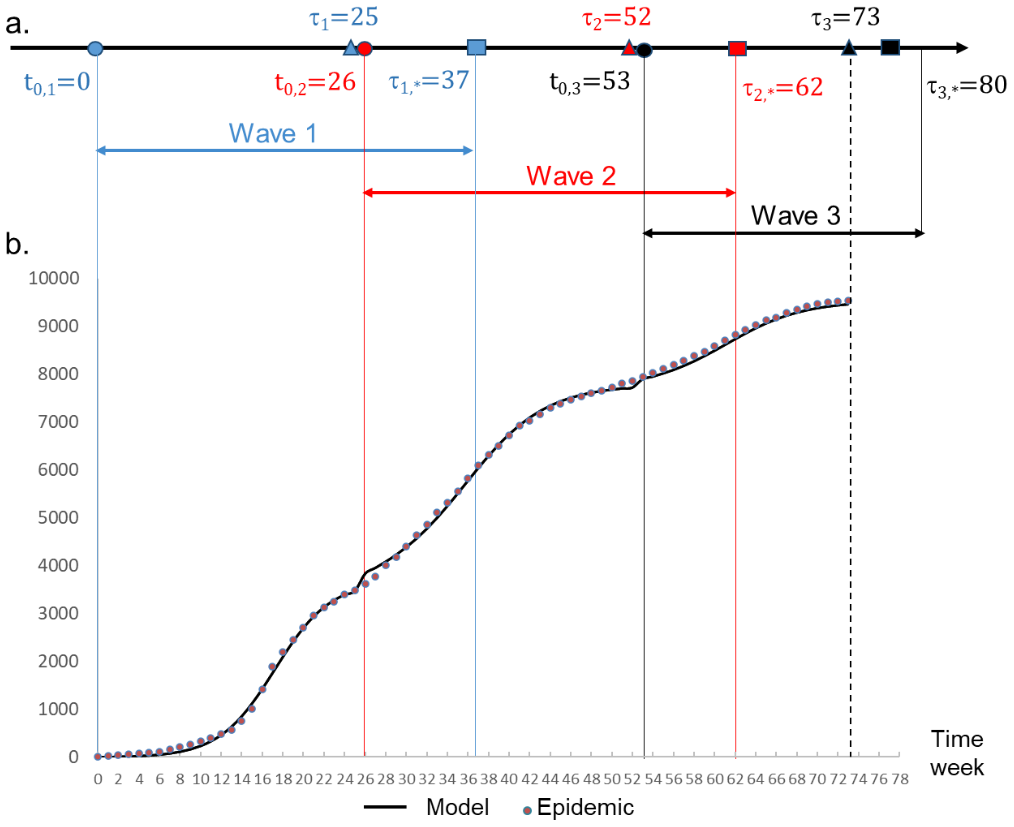

Figure S4. Temporal characteristics of the 2011-2012 HFMD epidemic. (**a**) Timeline parameters. t_0 is the beginning of a wave. The time τ corresponds to the last known case for a given wave. The time τ_* is an estimation of τ obtained by the model (equation 3). Beginning (t=0 in the model) of wave 1 was set at week 16 (supplemental Table 1). (**b**) Evolution of cumulative cases. Dots are associated to the observed values Y(t(i)). The solid curve is the theoretical number of cumulative cases $X_G(t)$.
